# Late-onset posttransplant Epstein-Barr virusrelated lymphoproliferative disease after cord blood transplantation for chronic active Epstein Barr virus infection: A case report

**DOI:** 10.1097/MD.0000000000029055

**Published:** 2022-03-25

**Authors:** Masayo Yamamoto, Motohiro Shindo, Takuya Funayama, Chihiro Sumi, Takeshi Saito, Yasumichi Toki, Mayumi Hatayama, Ken-Ichi Imadome, Yusuke Mizukami, Toshikatsu Okumura

**Affiliations:** a *Division of Metabolism and Biosystemic Science, Gastroenterology and Hematology/Oncology, Department of Medicine, Asahikawa Medical University, Asahikawa, Japan,*; b *Division of Advanced Medicine for Virus Infections, National Center for Child Health and Development, Tokyo, Japan.*

**Keywords:** Epstein-Barr-virus-related hemophagocytic lymphohistiocytosis, Epstein-Barr-virus-related lymphoproliferative disease, hematopoietic stem cell transplantation, late-onset posttransplant lymphoproliferative disease, posttransplant lymphoproliferative disease

## Abstract

**Introduction:**

Posttransplant lymphoproliferative disease (PTLD) is a critical complication of hematopoietic stem cell transplantation (HSCT). PTLD is classified into early and late-onset PTLDs. In post-HSCT patients, late-onset PTLD is rare, particularly PTLD after HSCT for Epstein-Barr virus (EBV)-related lymphoproliferative disease. Here, we report the case of a patient diagnosed with late-onset EBV-related hemophagocytic lymphohistiocytosis (HLH), that of PTLD, after HSCT for chronic active EBV infection (CAEBV), that of EBV related lymphoproliferative disease, probably because of EBV reactivation.

**Patient concerns and diagnosis:**

A 22-year-old woman with abdominal fullness visited our hospital. Blood examination showed pancytopenia with atypical lymphocytes, liver dysfunction, and elevated lactate dehydrogenase level. In contrast, bone marrow aspiration showed slight hemophagocytosis with increased natural-killer cells (NK cells). As serum antibodies against EBV were atypical, we calculated the EBV-DNA level in peripheral blood and this level was significantly high. EBV was infected with NK cells, and EBV's monoclonality in NK cells was confirmed. Thus, the patient was diagnosed with CAEBV

**Interventions and outcomes:**

The patient received chemotherapy and cord blood cell transplantation (CBT); CAEBV was well controlled. Approximately 6years from CBT for CAEBV, she visited our hospital because of fever. Blood examination revealed pancytopenia with atypical lymphocytes, liver dysfunction, and elevated lactate dehydrogenase level. In contrast, bone marrow aspiration showed hemophagocytosis with increased B and T cell counts without increased NK cell count. Additionally, serum antibody titers against EBV were atypical, and the EBV-DNA level in the peripheral blood was high. EBV was infected with only B cells, and EBV's monoclonality was confirmed. A more detailed analysis indicated that EBV-specific cytotoxic T lymphocytes were inactive. Therefore, she was diagnosed with late-onset EBV-related HLH. She received extensive treatment, but EBV-related HLH did not improve. Finally, she died about 3 weeks after diagnosis.

**Conclusion:**

PTLD, including HLH, is a life-threatening complication after transplantation, including HSCT. To our knowledge, this is the first case of late-onset EBV-related HLH after CBT for CAEBV. Late-onset PTLD has an indolent clinical course, but our patient's disease course was extremely aggressive. Therefore, late-onset EBV-related PTLD may be life-threatening.

## 1. Introduction

Posttransplant lymphoproliferative disease (PTLD) is life-threatening transplantation complication, including hematopoietic stem cell transplantation (HSCT),^[1-5]^ classified into early and late-onset PTLDs. Early-onset PTLD occurs within 2 years after transplantation,^[2-5]^ and it is mostly associated with Epstein-Barr virus (EBV)-infected B cells.^[2-4]^ However, late-onset PTLD may originate from T rather than B cells and is occasionally unassociated with EBV.^[2,3]^ Immunological deterioration induced by immunosuppressants is a major risk factor for late-onset PTLD.^[3-5]^ In patients after solid organ transplantation (SOT), late-onset PTLD is often reported,^[3-5]^ but it is rare in patients after HSCT. To the best of our knowledge, this is the first report of late-onset EBV-related hemophagocytic lymphohistiocytosis (HLH), that is, PTLD, for > 2years after cord blood cell transplantation (CBT) for chronic active EBV infection (CAEBV).

## 2. Case report

A 22-year-old woman with abdominal fullness visited our hospital. She had a history of mosquito bite hypersensitivity from childhood. She reported no other symptoms indicating infections, such as infectious mononucleosis (IM). Blood examination showed pancytopenia with atypical lymphocytes in peripheral blood, liver dysfunction, elevated lactate dehydrogenase (LDH) level, elevated serum ferritin level, and elevated soluble interleukin-2 receptor level. Bone marrow aspiration showed slight hemophagocytosis with increased number of natural- killer cells (NK cells). Computed tomography (CT) revealed hepatomegaly and splenomegaly, whereas positron emission tomography-computed tomography (PET-CT) revealed no uptake of fluorodeoxyglucose in the liver and spleen. Serum antibody titers against EBV were examined. We considered that these results were atypical because EBV-early antigen- diffuse and restricted antibody IgG was positive (80 times) and EBV nuclear antigen was weakly positive (10 times). Thus, we calculated the EBVDNA level in the peripheral blood, and this level was extremely high (1,100,000 copies/10^6^ cells). The monoclonality of EBV in the peripheral blood was confirmed through Southern blotting,^[6]^ and a significant amount of EBV-DNA was detected in the NK-cell fraction (4,600,000 copies/μg DNA). The monoclonality of EBV in NK cells was confirmed through Southern blotting. Thus, the patient was diagnosed with CAEBV of the NK-cell type. Consequently, she received immune cooling therapy (prednisolone+ cyclosporine: cyclosporine+etoposide); however, the treatment was ineffective. Afterward, she received multidrug chemotherapy; CAEBV improved, and EBV-DNA in the peripheral blood decreased. After chemotherapy, she received CBT for a radical cure. She had graft-versus-host disease because CBT and received immunosuppressants. Approximately 1 year after CBT, graft-versus-host disease improved, and immunosuppressants were discontinued. After CBT, the EBV-DNA load did not increase, and her mosquito bite hypersensitivity disappeared. Then, her EBV-viral capsid antigen antibody (EBV-VCA) IgG was positive. Moreover, the EBV-DNA level did not increase, indicating that CAEBV was well controlled. EBV antibody titers were not checked regularly for more than approximately 1 year after CBT. However, she visited our hospital regularly thereafter.

**Figure 1.**
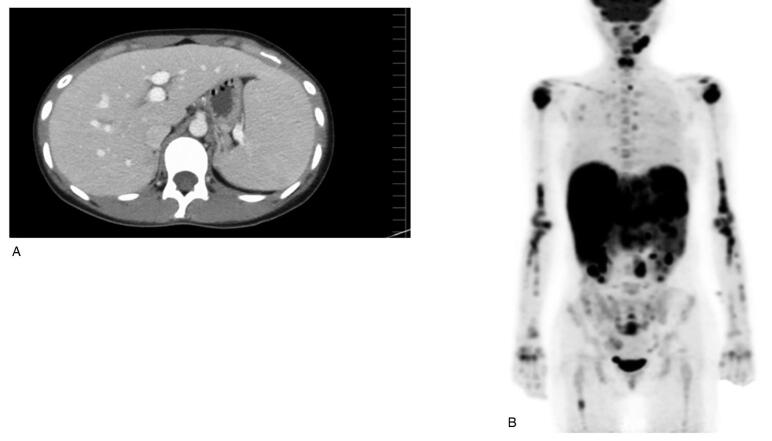
Image findings on admission. Enhanced computed tomography (A) showed hepatomegaly and splenomegaly. Positron emission tomographycomputed tomography (B) showed considerable uptake of fluorodeoxyglucose in the liver, spleen, lymph nodes of the entire body and bone.

**Figure 2.**
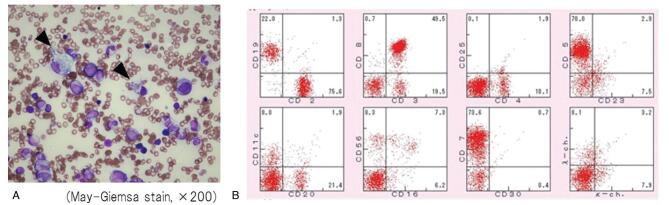
Findings of bone marrow aspiration. (A) Finding of bone marrow smear. Bone marrow was almost normocellular and there was no morphological abnormality including lymphocytes. Macrophages were increased and hemophagocytosis was noted (arrow). (B) Flow cytometric analysis of bone marrow cells. T cells were increased and CD8 positive T cells were more than CD4 positive T cells. B cells were also increased without the bias of the light chain. NK cells were not increased.

**Figure 3.**
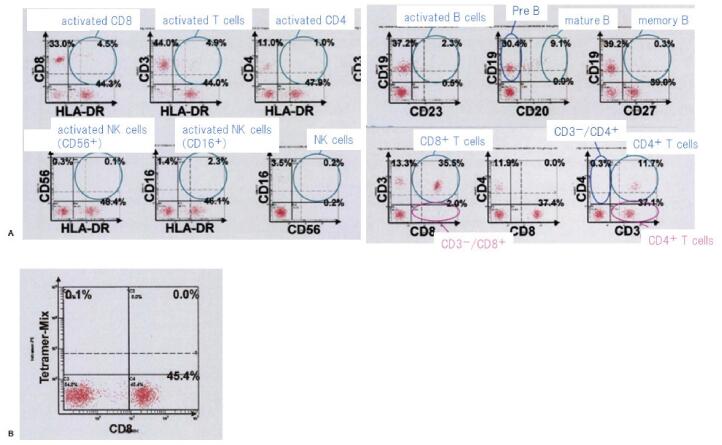
Detailed analysis via identifying EBV-infected cells in the peripheral blood. (A) Detailed analysis via flow cytometry. There were more CD8-positive T cells than CD4-positive T cells; but few activated CD8 positive T cells. B cells were increased, but they were not activated in this analysis. NK cells were not increased. (B) Result of flow cytometry analysis performed to identify EBV-specific cytotoxic T lymphocytes (CTL). We could detect EBV-specific CTL constrained by HLA-A*2402 tetramers mounted with five different EBV antigen peptides (derived from BMLF1, BMRF1, EBNA3A, EBNA3B, and LMP2) in this analysis. EBV-specific CTL was not activated. EBV = Epstein-Barr virus.

Approximately 6 years after CBT for CAEBV, she consulted a clinic for a fever. At the clinic, her blood examination revealed pancytopenia and liver dysfunction, and CT showed hepatomegaly and splenomegaly (Fig. 1A). Then, she was referred to our hospital (day 1). Blood examination at our hospital revealed pancytopenia with atypical lymphocytes, liver dysfunction, elevated serum ferritin level, elevated LDH level, and highly elevated soluble interleukin-2 receptor level (Table 1). PET-CT on day 4 revealed increased fluorodeoxyglucose uptake in the liver, spleen, and lymph nodes of the whole body and bone (Fig. 1B). Bone marrow aspiration showed slight hemophagocytosis with more B and T cells without increased NK cells (Fig. 2), and the rate of donor cell engraftment in the bone marrow was 100%. Serum antibody titers against EBV were atypical, representing both uninfected and already infected, because EBV early antigen-diffuse and restricted antibody IgG was slightly positive whereas EBV-VCA IgG was positive (Table 1). Then, we suspected PTLD, and evaluated the EBVDNA level in the peripheral blood; this level was high (Table 1). EBV's monoclonality in the peripheral blood was confirmed through Southern blotting. To clarify which cells were infected with EBV, we used the same method as that used when she was diagnosed with CAEBV.^[6]^ EBV-DNA was detected in the B cell fraction but not in other fractions. A more detailed analysis using flow cytometry revealed more CD8-positive than CD4-positive T cells but few activated CD8-positive T cells. B cells infected by EBV were increased, but they were not activated in this analysis (Fig. 3A). Flow cytometry showed that EBV-specific cytotoxic T lymphocytes (CTL) were inactive (Fig. 3B). As a result, we suspected late-onset EBV-relative PTLD or IM by primary infection of EBV after CBT. However, we could not distinguish them completely because we did not regularly calculate the titers of antibody against EBV and did not know her antibody status against EBV before this episode. However, because EBV-VCA IgG was positive and EBV-specific CTL was inactive, we suspected late-onset EBV-related HLH, that of PTLD, rather than IM.

**Table 1:** Laboratory data on admission.

WBC		1550	/μL	TP	7.3	g/dL	PT-%	67	%	sIL-2R	14393.0	U/mL
	Neu	47.0	%	Alb	3.8	g/dL	PT-INR	1.15
	Lym	39.0	%	T-bil	2.8	mg/dL	APTT	46.4	sec	EBV VCA IgG	320	times
	Mono	8.0	%	D-bil	0.3	mg/dL	Fib	275	mg/dL	EBV VCA IgM	< 10	times
	Eos	0.0	%	ALP	633	IU/L	D-dimer	1.76	mg/dL	EBV EADR IgG	40	times
	Baso	0.0	%	AST	343	IU/L	AT-III	92	%	EBV EBNA	40	times
	At-lym	6.0	%	ALT	187	IU/L				EBV DNA	7,400	copies/10^6^ cells
RBC		204 × 10^4^	/μL	LDH	1816	IU/L	Fe	33	μg/dL
Hb		9.4	g/dL	γ-GTP	65	IU/L	UIBC	254	μg/dL	HBs Ag	(-)
Ht		28.8	%	BUN	16.0	mg/dL	TIBC	287	μg/dL	HCV Ab	(-)
MCV		89.4	fL	Cre	0.56	mg/dL	Ferritin	2677.7	ng/mL	HAV Ab IgM	(-)
MCH		29.2	pg	Na	135	mmol/L				HEV IgA	(-)
MCHC		32.6	%	K	4.6	mmol/L	IgG	1868.5	mg/dL	CMV-C7HRP	(-)
Plt		3.8 × 10^4^	/μL	Cl	102	mmol/L	IgA	174.7	mg/dL
			Ca	8.6	mg/dL	IgM	162.1	mg/dL
			CRP	6.02	mg/dL

γ-GTP = γ-glutamyl transpeptidase, Alb = albumin, ALP = alkaline phosphatase, ALT = alanine aminotransferase, APTT = activated partial thromboplastin time, AST = aspartate aminotransferase, AT-III = antithrombin III, At-lym = atypical lymphocytes, Baso = basophils, BUN = blood urea nitrogen, CMV-C7HRP = cytomegalovirus pp65 antigen, Cre = creatinine, CRP = C-reactive protein, D-Bil = direct bilirubin, EBV EADR IgG = EBV-early antigen-diffuse and restricted antibody immunoglobulin G, EBV EBNA = EBV-nuclear antigen, EBV VCA IgG = EBV-viral capsid antigen antibody immunoglobulin G, EBV VCA IgM = EBV-viral capsid antigen antibody immunoglobulin M, Eos = eosinophils, Fe = serum iron, Fib = fibrinogen, HAV Ab IgM = hepatitis A virus antibody immunoglobulin M, Hb = hemoglobin, HBsAg = hepatitis B surface antigen, HCV Ab = hepatitisC virus antibody, HEV IgA = hepatitis E virus immunoglobulin A, Ht = hematocrit, IgA = immunoglobulin A, IgG = immunoglobulin G, IgM = immunoglobulin M, LDH = lactate dehydrogenase, Lym = lymphocytes, MCH = mean corpuscular hemoglobin, MCHC = mean corpuscular hemoglobin concentration, MCV = mean corpuscular volume, Mono = monocytes, Neu = neutrophils, Plt = platelets, PT = prothrombin time, PT-INR = prothrombin time-international normalized ratio, RBC = red blood cells, sIL-2R = soluble interleukin-2 receptor, T-Bil = total bilirubin, TIBC = total iron binding capacity, TP = total protein, UIBC = unsaturated iron binding capacity, WBC = white blood cells.

**Figure 4.**
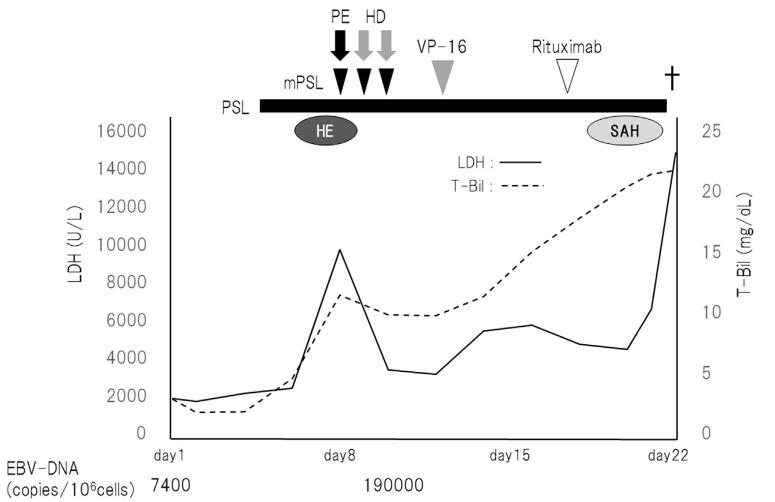
Clinical course after admission. The patient received prednisolone (PSL), but her total bilirubin (T-Bil) and lactate dehydrogenase (LDH) levels increased rapidly. Furthermore, she had symptom associated with hepatic encephalopathy (HE); therefore, we administered methylprednisolone (mPSL) pulse and plasma exchange therapy (PE) according to fulminant hepatitis. After PE, her condition improved and T-Bil and LDH levels decreased, so we switched to hemodialysis (HD) from PE. Because the level of EBV-DNA in the peripheral blood increased rapidly, we administered etoposide (VP-16). Nevertheless, T-Bil and LDH levels increased again. At this point, her EBV-infected cells were identified as B cells, and rituximab was administered. Among these treatments, her condition was complicated by subarachnoid hemorrhage, and she died on day 22. EBV-DNA = Epstein-Barr virus DNA.

The clinical course of this episode is shown in Figure 4. PETCT results showed PTLD, and we administered PSL (1 mg/kg/d) from day 5. After starting PSL, blood examinations on day 8 showed rapidly elevated total bilirubin (T-Bil) and LDH levels, and coagulation tests worsened. The patient met the diagnosis criteria of disseminated intravascular coagulation. Furthermore, she had symptoms possibly associated with hepatic encephalopathy; thus, she received methylprednisolone pulse (1 g/d) from days 8 to 10 and plasma exchange therapy (PE) according to fulminant hepatitis on day 8. After PE, the symptoms associated with hepatic encephalopathy improved and T-Bil and LDH levels decreased; hence, the blood purification therapy was changed from PE to hemodialysis and hemodialysis was performed on days 9 and 10. Because EBV-DNA in the peripheral blood increased during these therapies, she received etoposide for HLH. T-Bil and LDH levels increased, and disseminated intravascular coagulation worsened. Because the infected cells of EBV were B cells, rituximab was administered on day 19. Despite extensive treatment, HLH did not improve. Furthermore, she had a consciousness disturbance and was diagnosed with subarachnoid hemorrhage on day 21. Unfortunately, she died on day 22, and a pathological autopsy was performed.

**Figure 5.**
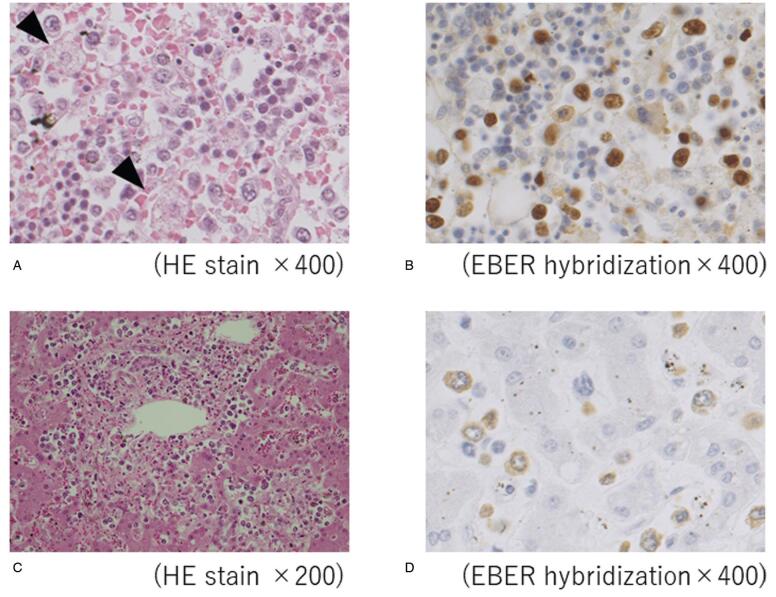
Pathological findings of bone marrow and liver from autopsy. (A) Finding of bone marrow with hematoxylin-eosin (HE) stain. Bone marrow was hypercellular, and large atypical cells were shown diffusely. Furthermore, macrophages increased and hemophagocytosis was revealed (arrow). (B) Finding of bone marrow with EBV-encoded small RNA (EBER) hybridization. The large atypical cells were positive for EBER. (C) Finding of the liver after HE staining. Centrilobular necrosis and large atypical cells were noted. (D) Finding of the liver with EBER hybridization. The large atypical cells were positive for EBER. EBV = Epstein-Barr virus.

Results showed large atypical cells in almost all organs, including the liver, spleen, lymph nodes, and bone marrow. The large atypical cells were positive for EBV- encoded small RNA in situ hybridization (Fig. 5). Furthermore, macrophages were increased, and hemophagocytosis noted in the liver, lymph nodes, and bone marrow (Fig. 5). Finally, her disease was confirmed as the late-onset EBV-related HLH, that of PTLD.

## 3. Discussion

PTLD is a life-threatening complication after transplantation, including HSCT.^[1-5]^ Most PTLD cases are associated with EBV-infected B cells.^[2]^ In patients undergoing HSCT, the risk of developing EBV-related PTLD is approximately 1% to 4%,^[1,2]^ and the mortality rate is approximately 80%.^[1]^ However, recently, the mortality rate has improved via novel approaches such as rituximab and EBV-specific CTL.^[1]^

PTLD is classified into 2 types by the onset of transplantation; early and late-onset PTLD. Early-onset PTLD develops within 2 years after transplantation,^[2-5]^ and is mostly associated with EBV; most cases originate from B cells.^[2-4]^ In patients undergoing SOT, transplants from an EBV-seropositive donor to an EBV-seronegative recipient increase the risk of early-onset PTLD.^[3]^ Early-onset PTLD is often more aggressive than lateonset PTLD, and the mortality rate is higher.^[2,4]^ However, lateonset PTLD mostly develops 2 years after transplantation.^[2-5]^ Late-onset PTLD originates from T rather than B cells,^[2,4]^ and almost half of the cases are associated with EBV.^[2-4]^ Late-onset PTLD shows more indolent clinical course than early-onset PTLD.^[2]^ Risk factors for late-onset PTLD are different from those of early-onset PTLD.^[2,7]^ The history of anti-rejection therapies, including immunosuppressant use, and EBV-seronegative recipients might be associated with late-onset PTLD in patients undergoing SOT.^[3,5]^ However, factors associated with it in patients undergoing HSCT remain obscure. The frequency of late-onset PTLD is unclear. It is often reported in patients undergoing SOT,^[3-5]^ but reports in post-HSCT patients are limited.^[2,8,9]^ We consider the present case as that of late-onset PTLD occurring 6 years after CBT for CAEBV. Although, lateonset PTLD has an indolent clinical course, our patient had an extremely aggressive clinical course, and she died approximately 3 weeks after being diagnosed with late-onset EBV-related HLH, that of PTLD. To our knowledge, this is the first case of late-onset EBV-related HLH occurring > 2 years after HSCT for CAEBV.

In post-HSCT patients, EBV infection can be caused by donor B-cells or EBV reactivation in the recipients, which can induce early-onset PTLD.^[9,11]^ Particularly, HSCT from an EBV-seronegative donor to an EBV-seropositive recipient can cause early-onset PTLD, which is triggered by EBV-infected cells in the recipient.^[11]^ Incidentally, donor CBT cells are assumed to be EBV negative^[11]^; thus, this case may have been that of HSCT from EBV-seronegative donor to EBV-seropositive recipient. After HSCT from an EBV-seronegative donor to an EBV-seropositive recipient, such as in this case, the titer of antibody against EBV and EBV viral load sometimes show negative findings indicating an EBV- uninfected status.^[11]^ If patients develop an EBV-related disease, including PTLD or IM there are 2 other possibilities 2 years after CBT, reactivation from latent cells and secondary primary infection.^[11]^ According to a previous report, patients who developed EBV-associated events after CBT, patients with persistent EBV-seropositivity after CBT showed typical PTLD and those with EBV-seronegativity after CBT developed IM.^[12]^ However, patients with indeterminate EBV status after CBT similar to our patient in this case, develop IM or PTLD.^[12]^ From these reports, antibody titers against EBV after CBT might help distinguish between PTLD by reactivation and IM by secondary primary infection. In the present case, because the antibody levels to EBV were not confirmed 1 year from HSCT, it was unavailable to distinguish between late-onset PTLD or IM.

IM is a typical disease caused by an acute EBV infection including secondary primary infection.^[13]^ It is an acute infectious disease characterized by fever, cervical lymphadenopathy, and pharyngitis.^[12]^ In primary EBV infection, including IM, EBV-VCA IgM is produced, and EBV-VCA IgG is produced after the first week of illness and persists for life.^[12]^ EBV nuclear antigen IgG is produced slowly and detected 2-3 months after IM onset, and its presence in the early clinical phase excludes acute primary EBV infection.^[12]^ In this case, the antibody titers against EBV were positive for EBV-VCA IgG, negative for EBV-VCA IgM, slightly positive for EBV nuclear antigen, and inconsistent with primary EBV infection. However, acute IM is characterized by abnormally high circulating CD8-positive T cells, called CTL,^[13-15]^ which is important for controlling EBV.^[10]^ In this case, flow cytometry (Figs. 3A and 3B) showed that CD8-positive T cells and EBV-specific CTL were inactive. From these findings, we diagnosed this case as late-onset EBV-related PTLD triggered by reactivation from latent EBV-infected cells. However, EBV might have infected the patient after CBT without IM development, and EBV-infected cells, which were infected at that point, might have caused late-onset EBV-related PTLD. Therefore, to distinguish between these possibilities, we should regularly evaluate the EBV-VCA IgG titers.

## 4. Conclusion

We reported the case of late-onset EBV-related HLH, that of PTLD, occurring 6 years after CBT for CAEBV. Because CBT donors were EBV-seronegative, EBV-reactivation from latent EBV-infected cells and secondary primary infection might have occurred. To confirm the clinical condition, anti-EBV antibody titers should be monitored regularly after CBT. In addition, late-onset EBV-related PTLD should be considered life-threatening.

## Acknowledgments

The authors thank Edanz (https://jp.edanz.com/ac) for editing a draft of this manuscript.

## Author contributions

**Conceptualization:** Masayo Yamamoto, Takuya Funayama, Chihiro Sumi, Takeshi Saito, Yasumichi Toki, Mayumi Hatayama.

**Data curation:** Masasyo Yamamoto, Motohiro Shindo, Takuya Funayama, Chihiro Sumi, Takeshi Saito, Yasumichi Toki, Mayumi Hatayama, Ken-Ichi Imadome.

**Investigation:** Masayo Yamamoto, Motohiro Shindo, Takuya Funayama, Chihiro Sumi, Takeshi Saito, Yasumichi Toki, Mayumi Hatayama, Yusuke Mizukami, Toshikatsu Okumura.

**Writing** - **original draft:** Masayo Yamamoto.

**Writing** - **review & editing:** Motohiro Shindo, Yusuke Mizukami, Toshikatsu Okumura.
